# Clinical and Genomic Investigation of an International Ceftriaxone- and Azithromycin-Resistant Shigella sonnei Cluster among Men Who Have Sex with Men, Montréal, Canada 2017–2019

**DOI:** 10.1128/spectrum.02337-21

**Published:** 2022-06-01

**Authors:** Christiane Gaudreau, Isabelle Bernaquez, Pierre A. Pilon, Alexandre Goyette, Nada Yared, Sadjia Bekal

**Affiliations:** a Microbiologie médicale et infectiologie, Centre Hospitalier de l’Université de Montréal (CHUM), Montréal, Quebec, Canada; b Département de microbiologie, infectiologie et immunologie, Université de Montréal, Montréal, Quebec, Canada; c Laboratoire de santé publique du Québec/Institut national de santé publique du Québec, Sainte-Anne-de-Bellevue, Quebec, Canada; d Direction régionale de santé publique, Centre intégré universitaire de santé et de services sociaux du Centre-Sud-de–l’île-de-Montréal, Montréal, Quebec, Canada; e Département de médecine sociale et préventive, Université de Montréal, Montréal, Quebec, Canada; f Microbiologie médicale et infectiologie, Hôpital Charles-Lemoyne, Greenfield Park, Quebec, Canada; University of Utah and ARUP Laboratories

**Keywords:** *Shigella sonnei*, outbreak, cluster, multidrug-resistant, ESBL, international, genomic, MSM, clinical, epidemiology

## Abstract

Multidrug-resistant (MDR) Shigella sonnei have become prevalent among men who have sex with men and have become a global public health concern. From June 2017 to April 2019, 32 men were infected with MDR S. sonnei acquired locally, in Montréal, which was suggestive of an outbreak. Antimicrobial susceptibility testing, whole-genome sequencing (WGS), phylogenetic analysis, antimicrobial resistance and virulence characterization, and association to international clusters were performed. The outbreak strain was ceftriaxone- and azithromycin-resistant due to the acquisition of *bla*_CTX-M-27_, and *mphA* and *ermB* genes, respectively, with reduced susceptibility to ciprofloxacin due to a single point mutation (*gyrA* S83L). One out of 27 patients treated with a fluoroquinolone experienced microbiological failure. Epidemiological evidence first supported by a rare unique MDR Shigella sonnei documented only in men in 2017 followed by similar pulsed-field gel electrophoresis profiles was confirmed by WGS. A core genome high-quality single-nucleotide variant (hqSNV)-based phylogeny found a median of 6 hqSNV differences among isolates. Virulence gene content was investigated, but no Shiga toxins were detected. An international cluster of highly related isolates was identified (PDS000019750.208) and belonged to the 3.7.29.1.4.1 S. sonnei genotype (Global III VN2.KH1.Aus). Genomic analysis revealed that this Montréal cluster was connected to other documented outbreaks in Australia, the United States, and the United Kingdom. This study highlights the urgent need for public health measures to focus on the prevention and the early detection of S. sonnei, since global transmission patterns of MDR strains is concerning and few antimicrobial treatment options are available.

**IMPORTANCE** Shigella sonnei, an important foodborne pathogen, recently became a frequent sexually transmitted agent involved in large and persistent outbreaks globally among men who have sex with men. Most strains also harbor several multidrug-resistant (MDR) determinants of particular concern. This study characterizes an outbreak strain at the source of an important MDR cluster identified in Montréal in 2017. Associations were made to many high-profile international outbreaks, and the causative S. sonnei lineage of these clusters was identified, which was not evident in past reports. The worldwide occurrence of this strain is of concern since treatment with antimicrobials like ceftriaxone and azithromycin may not be effective, and rare microbiological failures have been documented in patients treated with ciprofloxacin. Our investigation highlights the threats of *Shigella* spp. infection and the necessity for antimicrobial susceptibility monitoring in order to mitigate S. sonnei’s impact on public health and to avoid transmission to other at-risk communities.

## INTRODUCTION

*Shigella* spp. are one of the most important causative agents of diarrhea worldwide (1, 2). *Shigella* spp. transmission is facilitated by a very low infectious inoculum capable of transmitting infection directly or indirectly from person to person, through the fecal-oral route. The four *Shigella* species (S. boydii, S. dysenteriae, S. flexneri, and S. sonnei) have evolved separately but convergently from distinct Escherichia coli lineages to become facultative intracellular human-adapted pathogens that cause similar syndromes (3). However, the disease severity and geographic distribution differ between species (4). In Canada and in other developed countries, S. sonnei infections have historically been associated with periodic food and/or water contamination, and with recent travel to places where the bacterium is endemic (5–8). However, most cases are now commonly detected among men who have sex with men (MSM) and have since caused large-scale outbreaks, sometimes spanning different continents (9–12). These outbreaks have often implicated particular S. sonnei lineages that have now been strongly associated with MSM (12) and are generally characterized as multidrug resistant (MDR; resistant to 3 or 4 of the 5 antibiotic classes recommended for *Shigella* spp. treatment) (9–21).

Although most *Shigella* spp. infections are self-limited, ciprofloxacin, azithromycin, and ceftriaxone are the first-line, while ampicillin and trimethoprim-sulfamethoxazole (TMP/SMX) are the second-line, antimicrobial treatments recommended for adult patients with severe infections or weakened immune systems (22). The World Health Organization included *Shigella* spp. on their priority pathogens list for research and development of new antibiotics due to the emergence of fluoroquinolone resistance (e.g., ciprofloxacin) (23, 24). This retrospective study characterizes a MDR S. sonnei outbreak strain collected in Montréal, describes the epidemiological information and treatment outcomes of the patients, and further extends the global transmission profile of this pathogen.

## RESULTS

### Epidemiological investigation.

**FIG 1:**
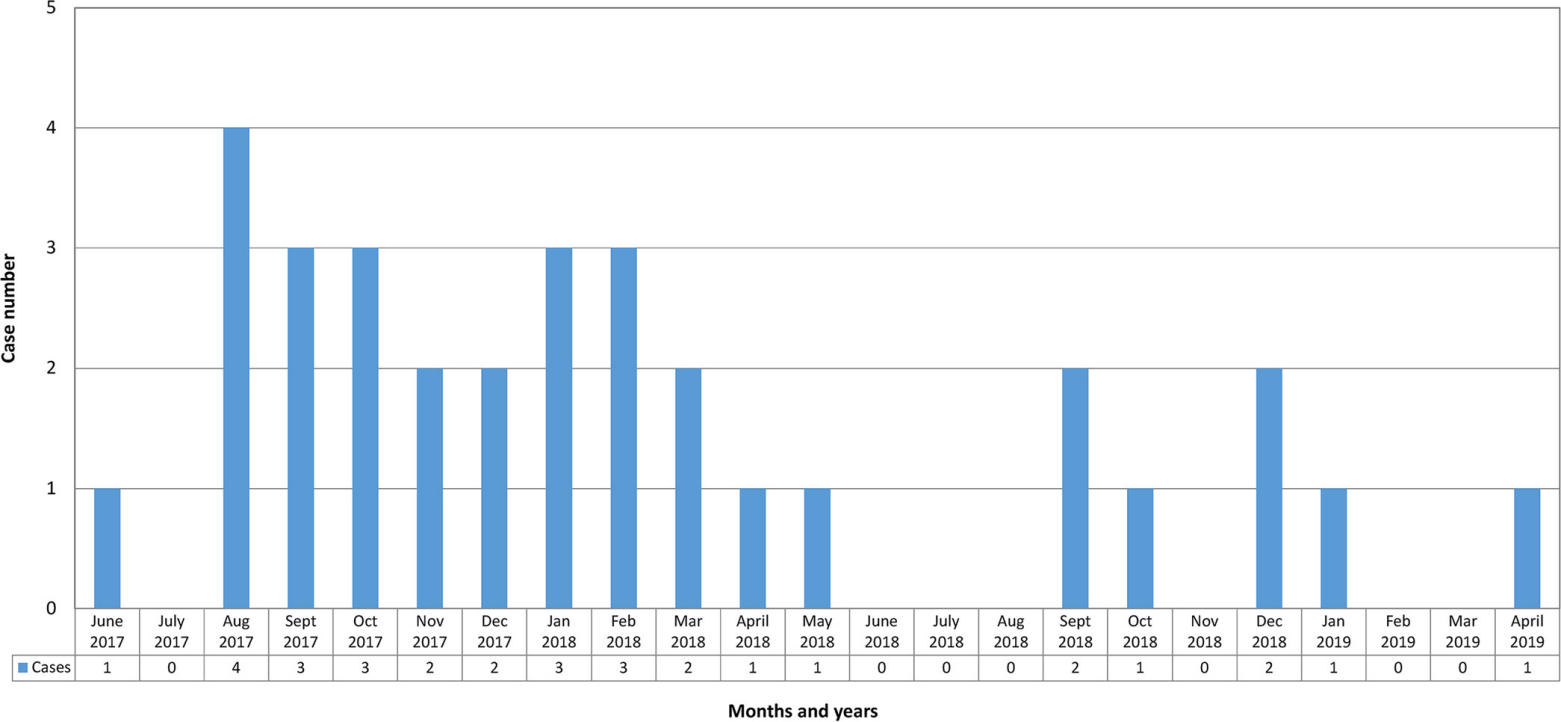
Epidemic curve of the 32 outbreak-related multidrug-resistant Shigella sonnei, Montréal, 2017–2019.

From June 2017 to April 2019, 32 men, 26 to 73 (median 40) years old, presented with an enterocolitis caused by a ceftriaxone- and azithromycin-resistant Shigella sonnei with reduced susceptibility to ciprofloxacin (based on the Centers for Disease Control and Prevention [CDC] criteria, but susceptible to this agent according to the Clinical and Laboratory Standards Institute (CLSI) and the European Committee on Antimicrobial Susceptibility Testing [EUCAST] criteria). Between January 2017 and December 2019 at the Centre Hospitalier de l’Université de Montréal (CHUM), no other Shigella sonnei presented this resistance profile out of a total of 109 patients. The epidemic curve was highly suggestive of an outbreak event (Fig. 1). Of the 32 men, 31 reported that they had sex with men; the sexual behavior of the other man was unknown. None of these patients had travelled outside Canada in the week before the onset of the enteric symptoms. Twenty-eight patients reported sexual contact prior to illness onset: 24 patients had anal intercourse, 11 patients had sexual contact with anonymous partners, and 5 patients (and one partner of another patient) reported visiting 2 different bathhouses. None of these 6 patients associated with bathhouses was the index case.

Of the 32 men, 12 were human immunodeficiency virus (HIV)-positive patients. The 12 HIV-positive men were all on antiretroviral treatment. All HIV-positive patients had CD4 cell counts that ranged from 270 to 710 (median 530) × 10^6^/L. Of the 12 HIV-positive patients, 10 had an HIV viral load of <20 copies/mL, 1 patient had a viral load of 314 copies/mL, and 1 patient had a viral load of 171,130 copies/mL. Nineteen patients were HIV-negative, and the HIV status was unknown for one patient. In the previous 6 years, 23 of these patients were diagnosed with sexually transmitted infections: 16 Treponema pallidum, 47 Neisseria gonorrhoeae, 31 Chlamydia trachomatis (including 2 lymphoma venereum), 4 *Shigella* spp., 2 Giardia lamblia, 3 hepatitis C, and 2 hepatitis B infections.

Of the 32 patients, two were food handlers and one worked in a healthcare facility. These 3 patients were respectively the 3rd, 4th, and 25th patients, and none of them was the index case of the cluster. None of the 32 patients worked in daycare centers. Thirty-one of the 32 patients presented the following symptoms: diarrhea (*n* = 31), abdominal pain (*n* = 20), fever (*n* = 12), nausea (*n* = 9), pus/mucus in stools (*n* = 6), blood in stool specimen (*n* = 7), and vomiting (*n* = 3). Five patients required hospitalization for their enteric infection. Twenty-five patients were treated with ciprofloxacin, out of which 14, 4, 5, and 2 patients were treated for 3, 5, 7, and 14 days, respectively. Two patients were treated with levofloxacin for 5 days, and one patient was treated with trimethoprim-sulfamethoxazole (TMP/SMX).

A patient consulted his primary care physician and reported up to 12 very loose nonbloody stools per day and abdominal cramps that lasted for a week. Microbiological analysis revealed that the patient’s stool was positive for S. sonnei. Antimicrobial susceptibility testing (AST) revealed that the isolate (SS075624) was susceptible only to ciprofloxacin, and the patient was treated with oral ciprofloxacin 1,000 mg daily for 3 days. About a week after the end of ciprofloxacin treatment, the repeat stool culture was positive for the same S. sonnei strain (SS079888), collected 26 days after the first positive stool culture. Overall, he had persistent symptoms for 6 weeks. Two repeat stool cultures were negative.

The two food handlers and the healthcare worker were excluded from their work pending 2 negative stool cultures done on different days after the end of their fluoroquinolone treatment. Fourteen other patients had either 1 (*n* = 8 patients) or 2 (*n* = 6 patients) negative stool cultures after fluoroquinolone treatment. To our knowledge, none of the other 31 patients had a prolonged or recurrent enteric disease. As part of the interventions to control the outbreak, bathhouses were visited to raise awareness among owners and community stakeholders, and enhanced environmental health measures, especially for high touch areas, were recommended. Community-based organizations that work with MSM were contacted to disseminate information on preventive measures.

### Antimicrobial susceptibility testing.

The 33 isolates were resistant to ampicillin, TMP/SMX and nalidixic acid (6 mm for the 3 agents), azithromycin (MICs > 256 mg/L), ceftriaxone (6 mm and 64–128 mg/L), cefixime (16–32 mg/L), and tetracycline (6 mm and 128–256 mg/L), but were susceptible to ertapenem (31–32 mm). These isolates had MICs of 0.12–0.25 mg/L to ciprofloxacin and were susceptible to this agent based on the CLSI (25) and EUCAST criteria (26) but were considered to have reduced susceptibility to ciprofloxacin with the CDC criteria (27). These S. sonnei isolates showed an extended spectrum beta-lactamase (ESBL)-positive phenotype, detected by ceftazidime, cefotaxime, and cefepime E-tests with and without clavulanic acid.

### PFGE typing and whole-genome sequencing (WGS)-based phylogeny.

**FIG 2:**
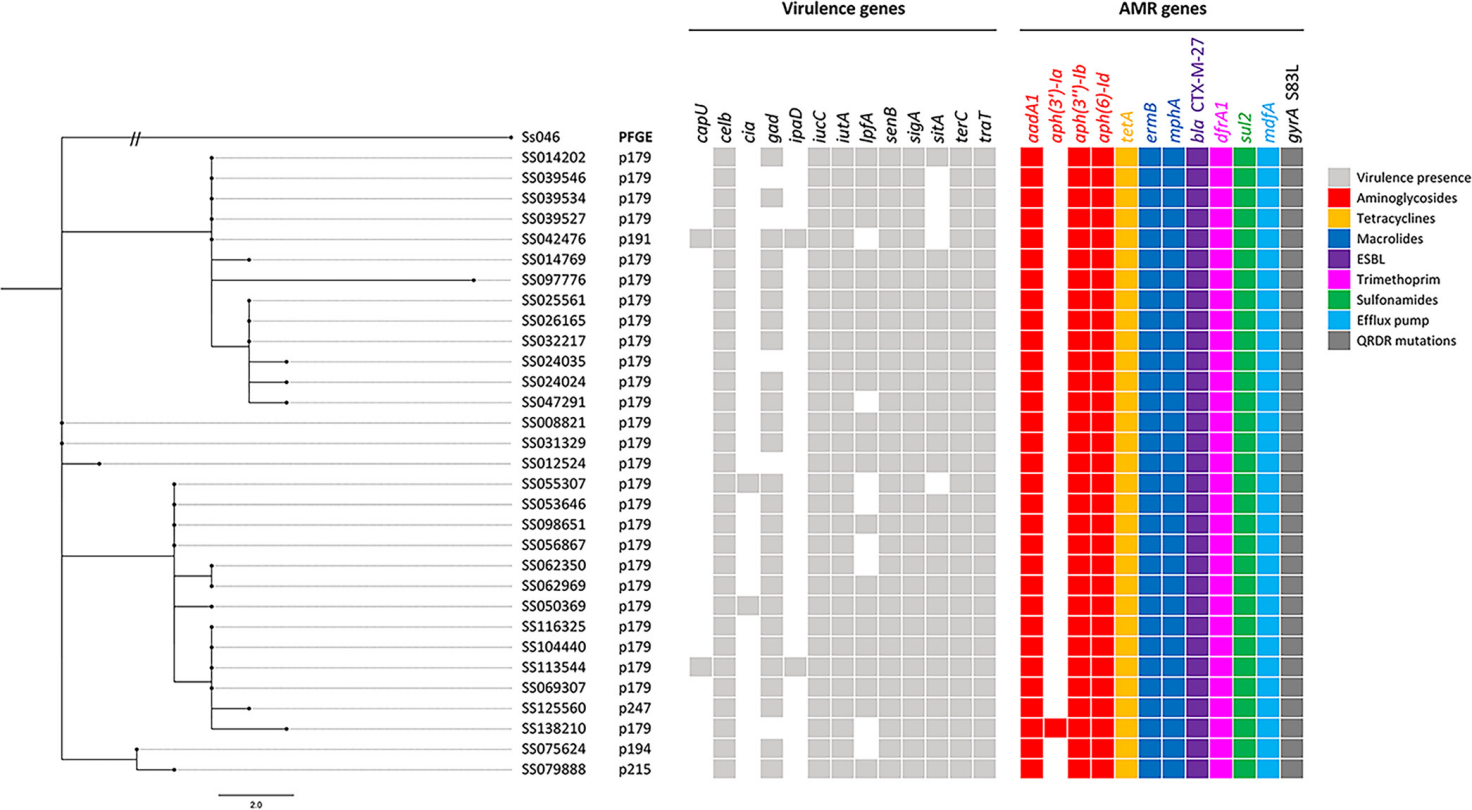
Maximum-likelihood phylogenetic tree and AMR- and virulence-associated profiles of the 31 sequenced Shigella sonnei cases related to an MSM outbreak in Montréal, 2017–2019. The phylogeny was based on 280 shared hqSNV positions from a shared genome of 3,534,922 bp determined by SNVPhyl for the sequenced Shigella sonnei isolates (*n* = 31) under study in relation to the reference chromosome CP000038.1 (illustrated as Ss046). The scale bar refers to the number of hqSNVs in function of the branch length, for the exception of Ss046, where the branch has been manually trimmed. The phylogenetic tree was produced using FigTree v1.4.3.

Pulsed-field gel electrophoresis (PFGE) revealed that 28 out of the 33 isolates had similar PFGE patterns designated as pulsovar 179, and the remaining isolates belonged to 5 related pulsovars (i.e., 191, 194, 197, 215, and 247) (data not shown). Phylogenetic analysis performed on the 31 sequenced S. sonnei isolates (from 30 different patients) confirmed that these isolates were genetically related. In fact, the isolates clustered all within the same clade and only differed from each other by 0–17 hqSNVs with a median and average pairwise distance of 6 hqSNVs (Fig. 2) in concordance with PFGE and epidemiological data. The genetic distances between the closest related isolates of different patients were ≤2 hqSNVs, with the exception of isolate SS097776, which had a genetic distance of ≥7 hqSNVs from the rest but was the last isolate collected in April 2019. Both isolates collected from the patient who demonstrated a clinical and microbiological failure were considered closely related (1 hqSNV difference) by SNVPhyl. This outbreak was declared multiprovincial by PulseNet Canada. Twenty-nine out of the 31 sequences were also included in a phylogenetic study of clinical S. sonnei sequences from the province of Quebec from August 2012 to December 2019, which puts this outbreak (comprising a total of 57 cases from Quebec) in a provincial context allowing the comparison with other circulating strains (28).

### Antimicrobial resistance determinants.

*In silico* AMR detection supported the AST results as all 31 sequenced S. sonnei isolates had acquired genes conferring resistance to TMP/SMX (*dfrA*1 and *sul*2), azithromycin (*ermB* and *mphA*), tetracycline (*tetA*), and ampicillin, ceftriaxone, and cefixime via an ESBL (*bla*_CTX-M-27_). Most isolates had acquired a total of 9 AMR-conferring genes, with the exception of SS138210, which acquired 10. A predicted resistance phenotype for streptomycin [*aadA1* and *aph(3″)-Ib]* and kanamycin [*aph(3′)-Ia* and *aph(6)-ld*] was also detected, but not routinely tested by AST as aminoglycosides are not clinically relevant for the treatment of *Shigella* spp. All isolates also possessed *mdfA*, which encodes a multidrug exporter. The resistance to nalidixic acid and the reduced susceptibility to ciprofloxacin according to the CDC criteria can be attributed to a single amino acid change (S83L) found in the quinolone resistance-determining region (QRDR) of the *gyrA* gene, as no *qnr* genes were identified. One out of the 27 patients treated with a fluoroquinolone exhibited a clinical and microbiological failure (diarrhea for 6 weeks and stool culture positive after ciprofloxacin treatment). Using ResFinder, it was found that this failure was unrelated to any additional unique known or unknown mutations detected in the QRDRs of the isolates recovered before (SS075624) and after (SS079888) treatment.

### Virulence content.

A total of 13 different virulence genes (*capU, celb, cia, gad, ipaD, iucC, iutA, lpfA, senB, sigA, sitA, terC*, and *traT*) were found in our dataset of 31 sequenced isolates. The virulence genes *celb* (colicin E2 enterotoxin), *gad* (glutamate decarboxylase), *iucC* (aerobactin synthetase), *iutA* (ferric aerobactin receptor), *lpfA* (long polar fimbriae), *senB* (plasmid-encoded enterotoxin), *sigA* (*Shigella* IgA-like protease homologue), *sitA* (iron transport protein), *terC* (tellurium ion resistance protein), and *traT* (outer membrane protein complements resistance) were present in most isolates (83.9–100%). The genes *capU* (hexosyltransferase homolog) and *ipaD* (invasion protein) were always present together, but were only found in 2 out of the 31 isolates. The *cia* gene (colicin ia) was equally present only in 2 out of the 31 isolates. Fig. 2 illustrates the SNVPhyl phylogeny and associated virulence- and AMR-conferring genes for each isolate.

### Global genotyping and clustering.

All 31 sequences from this study were included in the NCBI Pathogen Detection Isolates Browser, and it was found that they belonged to a distinct single nucleotide variant (SNP) cluster (PDS000019750.208) comprising 465 isolates. The distance between these isolates ranged from 0 to 49 SNPs, with an average distance of 16 SNPs. Excluding the isolates under study, the other 434 isolates were collected by various jurisdictions: Australia (*n* = 44), Belgium (*n* = 21), Canada (*n* = 28), New Zealand (*n* = 2), Spain (*n* = 1), Switzerland (*n* = 1), United Kingdom (*n* = 31), and United States (*n* = 306). The Swiss isolate was characterized in a study of CTX-M-producing S. sonnei in Switzerland (19). Twenty-four of the U.S. isolates were collected during a 2018 outbreak in a retirement community in Vermont (17). The Spain isolate was characterized as MSM associated (18). In addition, 35 out of the 44 Australian isolates included in this SNP cluster were associated to a prolonged MDR S. sonnei outbreak harboring *bla*_CTX-M-27_ that occurred from June 2019 to March 2020 (11). The other 7 and 2 Australian isolates were associated with circulating MDR strains collected from 2016–2017 (15) and 2018 (16), respectively. According to a global S. sonnei genotyping initiative (12), these 9 documented isolates fell into the hierarchical 3.7.29.1.4.1 genotype (lineage 3; clade 3,7; subclade 3.7.29), designated “Global III VN2.KH1.Aus,” alongside 28 United Kingdom isolates collected from 2016–2019, which were also included in our SNP cluster.

## DISCUSSION

In this 22-month long cluster of S. sonnei infection, 31 patients were MSM, 12 were HIV-positive, and 23 patients had other sexually transmitted diseases than HIV and no food or water source was identified, which strongly suggested sexually transmitted infections. Cluster isolates were ceftriaxone- and azithromycin-resistant and showed reduced susceptibility to ciprofloxacin (with the CDC criteria). The unique rare antimicrobial profile, the PFGE profiles’ similarity, and the genomic clustering confirmed that these 32 men were from an outbreak.

Genomic studies using SNVPhyl on well-characterized MSM-related Shigella sonnei outbreaks are limited; however, a California study has characterized an S. sonnei outbreak of 42 cases differing by 0 to 11 SNPs from each other by using similar genomic methods (20). In addition, an Australian MSM-related S. sonnei cluster of 35 ESBL isolates had a median pairwise distance of 3 SNPs (interquartile range 2 to 4 SNPs) using similar genomic approaches (11). Therefore, a 17 hqSNV diversity (median pairwise distance of 6 hqSNVs) in the outbreak under study follows expected genetic differences observed in epidemiologically supported *Shigella* spp. outbreaks. However, the larger diversity per number of cases observed in this study could have arisen from a longer time span (22 months) than the other studies and that our analysis only included patients who had stool cultures at the CHUM. In addition, differences in the phylogenetic parameters and datasets used in regard to the reference-based core genome analysis could also have an impact on SNV distance comparisons.

Virulence gene presence and frequencies for our S. sonnei cluster were considered normal, since many hallmarks of S. sonnei were detected, such as *celb*, *gad*, *lpfA*, *senB*, and *sigA* (29, 30). The low detection frequency of some of the virulence genes (*capU* and *ipaD*) can be attributed to their location on the large virulence plasmid, which can be highly unstable in storage or in laboratory media and is therefore commonly lost upon subculturing (31). Therefore, this plasmid is often missing from WGS data. Colicin (*cia*) is known to be produced by E. coli against competitors as a defense mechanism and was most likely acquired by a plasmid due to its sporadic presence in the phylogenetic tree. The absence of Shiga toxin virulence genes (*stx*), which had been detected in a Californian S. sonnei outbreak (20) and in Haïti travel-related Shigella flexneri cases in Montréal, (32) was notable.

All 31 sequenced isolates belonged to the PSD000019750.208 SNP cluster (*n* = 465) generated by NCBI’s Pathogen Detection Isolate Browser. Thirty-five of the 44 Australian isolates included in this cluster were associated with a prolonged MDR S. sonnei outbreak harboring *bla*_CTX-M-27_ that occurred from June 2019 to March 2020 in Victoria, Australia (11). The Australian isolates in this cluster also shared an MDR profile with a cluster of MSM-associated S. sonnei isolates from the United Kingdom dating back from March to November 2018 (9). Public Health England found that isolates belonging to this cluster also clustered with isolates from MSM patients from the United States. According to Ingle et al. (11), 2 S. sonnei isolates recovered from 2 returned travelers from Southeast Asia also exhibited a similar MDR profile and descended from the same lineage, suggesting that this strain was already circulating in Southeast Asia in 2017. Incidentally, as presented by this study, this strain was also circulating in Canada at this time. None of the 32 patients included in this study had recent international travel, although it is possible that some of these patients may have had contact with international tourists; however, no known contacts were declared in the patient charts. Another 24 cases from the United States were identified in this SNP cluster and belonged to a 2018 outbreak in a retirement community in Vermont (17). The outbreak was linked to an infected food handler. Microbiological failure after ciprofloxacin treatment was reported in 1 of the 15 individuals infected with the outbreak strain. The person who happened to be a staff member was infected with a susceptible S. sonnei strain (MIC = 0.12 mg/L) according to CLSI and EUCAST criteria and treated with ciprofloxacin, similar to our case. In previous studies, it was shown that nalidixic acid disk diameters or MIC breakpoints or both were reliable to document the reduced susceptibility to ciprofloxacin (9, 33). However, for now, the AST interpretation criteria of CLSI and EUCAST should not be considered for revision because, so far, there is not enough data on microbiological failures documented with such isolates.

The 3.7.29.1.4.1 S. sonnei genotype, namely, Global III VN2.KH1.Aus, was inferred to our cluster through the characterizations made on the published datasets that were included within our SNP cluster. Lineage 3 is the most common of the five deep-branching S. sonnei lineages, and the 3.7 clade is the most widely distributed and accounts for most S. sonnei infections in all 6 continents, with the exception of Latin America (12). This clade has also been shown to have a high concentration of azithromycin and extended spectrum cephalosporin resistance determinants (12). As far as we know, the earliest isolate included in the 3.7.29.1.4.1 genotype was isolated in Australia in 2016, but seems to have emerged from the 3.7.29.1.4 (VN2.KH1) genotype originating from the Kanh Hoa province in Vietnam while occasionally acquiring an ESBL, *ermB*, and *mphA* gene (12).

In our cluster, the S. sonnei isolates have a unique rare antimicrobial resistance pattern that had not been documented from 2009 to 2016 and in 2020 at the CHUM. In the United States and in Canada, a high percentage of *Shigella* spp. are ampicillin- and TMP/SMX-resistant (14, 34). Worldwide, S. sonnei isolates have long been resistant to certain antibiotics due to the acquisition and maintenance of small MDR plasmids (containing *sul2* and *tetA*) and transposons (containing *dfrA1* and *aadA1*) (12). Concerningly, infections with *Shigella* spp. that show resistance to ciprofloxacin, azithromycin, or both are now increasing in patients with risk factors for these infections (14, 34). An official health advisory from the CDC described the emergence of *Shigella* spp. with reduced susceptibility to ciprofloxacin with MICs of 0.12–1 mg/L (27), while MICs of **≤**0.25 are so far defined as susceptible by CLSI and EUCAST (25, 26). In previous reports, most ceftriaxone-resistant *Shigella* spp. isolates were carrying an ESBL (33), most having acquired *bla*_CTX-M-27_ (9, 11, 21). However, many different ESBL genes have been identified in S. sonnei (35), and there seems to be no association between specific genotypes and particular ESBL (12), meaning that these genes have been acquired independently, perhaps due to the fitness cost associated with the maintenance of such genes in the absence of antibiotic pressure (36).

In the United States, 2.7% of 474 *Shigella* spp. were resistant to ceftriaxone in 2018 (37). At the CHUM, no ceftriaxone-resistant *Shigella* spp. were documented from 2009 to 2020 in 417 patients (unpublished data) other than the 32 patients infected with the Shigella sonnei described in this study. However, many countries have noted an increase in 3rd generation cephalosporin resistant S. sonnei isolates: 31.6% in 2012 to 64.3% in 2015 in China (38), and 3.8% in 2016 to 37.5% in 2019 in Switzerland (19), among others (12, 36). Resistance to all recommended oral antibiotics have also appeared in multiple occasions in isolates carrying ≥3 QRDR mutations, an ESBL, and *mphA* (12). According to Hawkey et al. (12), all triple-resistant (azithromycin, ciprofloxacin, and 3rd generation cephalosporins) S. sonnei isolates detected in their study emerged from the 3.6.1.1 genotype (CipR) and appeared as early as 2014. Concerningly, the 3.6.1.1.2 genotype (CipR.MSM5), which harbors triple QRDR mutations and *mphA*, was the dominant genotype found in S. sonnei surveillance data from Australia, England, and the United States in 2019 (12). Should this strain acquire a stable mobile element with an ESBL and circulate in the MSM community, urgent public health measures will be required to prevent its global transmission.

Owing to the microbiological and/or clinical failures observed for ciprofloxacin-nonsusceptible strains and the recently abundant quinolone-resistant lineages, less antimicrobial agents are available to treat severely ill or immunosuppressed patients (14, 33, 34). Infections with MDR *Shigella* spp. may be of longer duration, have higher costs, and cause more morbidity than expected (33, 34). According to Public Health England, suboptimal responses to ciprofloxacin treatment have been observed for strains with the single QRDR *gyrA* S83L mutation, and symptoms (especially diarrhea) may be prolonged beyond 7 days (9).

Patients should avoid sex during symptomatic infections until 2 negative stool cultures or convalescence for many weeks (34). Patients with a *Shigella* spp. infection should be advised about preventive practices such as frequent hand washing with soap and precautions when manipulating food and water (34). MSM should use barriers during oral, anal, and genital sex, in addition to washing their genitals, anus, toys, and hands with soap before and after sex (34).

Bacterial stool cultures should be requested for patients with diarrhea, and AST of *Shigella* spp. isolates should be done (34). The CLSI recommend ampicillin, TMP**/**SMX, and ciprofloxacin testing for all *Shigella* spp. isolates (25). The MDR status of the Shigella sonnei isolates involved in the outbreak described in this study would not have been documented if susceptibility testing were limited only to ampicillin, TMP/SMX, and ciprofloxacin. With the standardization of azithromycin susceptibility for *Shigella* spp. established by CLSI in 2021 (25) and the use of this agent as an antimicrobial treatment, the routine testing and report of azithromycin susceptibility for all *Shigella* spp. isolates would be useful. In some centers with frequent MDR *Shigella* spp., the testing of ceftriaxone should also be considered for all *Shigella* spp. isolates and routinely reported if no oral antimicrobial treatments are available.

More data are needed on the clinical and microbiological responses of patients infected with *Shigella* spp. that show reduced susceptibility to ciprofloxacin (CDC criteria) and treated with a fluoroquinolone, as suggested (17). Only one isolate per patient and per outbreak should be included in order not to overestimate percentages of antimicrobial resistance. Urgent public health measures are therefore required to enhance the surveillance of MDR S. sonnei in order to put an early end to a potentially wide-spanning transmission event and to detect the potential emergence of extensively drug-resistant S. sonnei, resistant to all recommended oral antimicrobials for the treatment of S. sonnei (10, 16). Studying the genetic environment of clinically relevant AMR genes is of interest to clinicians and public health stakeholders. Genomic analysis can help to elucidate resistance mechanisms and identify new antibiotic targets, while providing insight into how these genes are maintained and disseminated, which can potentially support outbreak investigations by suggesting possible sources of contamination and/or mode of transmission.

## MATERIALS AND METHODS

### Bacterial isolates.

Stool cultures for *Shigella* spp. were grown on MacConkey, *Shigella*-Salmonella, and Xylose-Lysine-Deoxycolate agars incubated at 35°C in ambient air for 48 h at the microbiological laboratory, at the CHUM. Genus and species identification and serogrouping were done respectively by biochemical tests (API 20E) and slide agglutination (14). All 33 Shigella sonnei isolated from 32 patients with their corresponding metadata are described in Table 1.

**TABLE 1:** Metadata and BioSample information for the 33 outbreak-related Shigella sonnei isolates from Montréal, 2017–2019

Sample name	Collection date (yyyy-mm)	Pulsovar	Accession	BioSample
SS008821	2017-07	179	SRS6890400	SAMN15300344
SS012524	2017-08	179	SRS6890393	SAMN15300345
SS014202	2017-08	179	SRS6890397	SAMN15300346
SS014769	2017-08	179	SRS6890388	SAMN15300347
SS016757	2017-08	179	NS^*a*^	NS
SS024024	2017-10	179	SRS6890399	SAMN15300348
SS024035	2017-10	179	SRS6890392	SAMN15300349
SS025561	2017-10	179	SRS6890398	SAMN15300350
SS026165	2017-10	179	SRS6890394	SAMN15300351
SS031329	2017-10	179	SRS6890395	SAMN15300352
SS032217	2017-11	179	SRS6890402	SAMN15300353
SS039527	2017-12	179	SRS6890404	SAMN15300354
SS039534	2017-12	179	SRS6890407	SAMN15300356
SS039546	2017-12	179	SRS6890389	SAMN15300355
SS042476	2017-12	191	SRS6890384	SAMN15300357
SS047291	2018-01	179	SRS6890379	SAMN15300358
SS050352	2018-01	197	NS	NS
SS050369	2018-02	179	SRS6890390	SAMN15300359
SS053646	2018-02	179	SRS6890385	SAMN15300360
SS055307	2018-02	179	SRS6890386	SAMN15300361
SS056867	2018-02	179	SRS6890381	SAMN15300362
SS062350	2018-03	179	SRS6890382	SAMN15300363
SS062969	2018-04	179	SRS6890383	SAMN15300364
SS069307	2018-05	179	SRS6890380	SAMN15300365
SS075624^*b*^	2018-05	194	SRS6890387	SAMN15300366
SS079888^*b*^	2018-06	215	SRS10172684	SAMN18140753
SS097776	2018-09	179	SRS6890406	SAMN15300367
SS098651	2018-09	179	SRS6890396	SAMN15300368
SS104440	2018-10	179	SRS6890408	SAMN15300369
SS113544	2018-12	179	SRS6890405	SAMN15300370
SS116325	2018-12	179	SRS6890391	SAMN15300371
SS125560	2019-01	247	SRS6890401	SAMN15300372
SS138210	2019-04	179	SRS6890403	SAMN15300373

a NS, not sequenced.

b Samples from the same patient.

### Antimicrobial susceptibility testing.

AST was performed by disk diffusion according to the CLSI guidelines or E-tests as recommended by bioMérieux, Marcy-l'Étoile, France. The diameters and MIC results were interpreted using the breakpoints of the CLSI 2021 guidelines (25). The AST was done on Mueller-Hinton agar with an inoculum of 0,5 McFarland, incubated at 35–37°C in ambient air for 16–18 h (25). The quality control isolates and their ranges obtained were those recommended by CLSI and by the manufacturer (25).

### Epidemiological investigation.

After review and approval of the Comité d'éthique de la recherche du CHUM concerning the patients and isolates data, the hospital’s charts, the Direction régionale de santé publique de Montréal’s investigation charts (standardized questionnaire), and the Shigella sonnei isolate studies of the 32 patients were reviewed. Clinical and microbiological failures were respectively defined as persistent diarrhea (3 loose stools or more daily) and stool culture positive for the same MDR Shigella sonnei strain after completion of the antimicrobial treatment. The epidemic curve is illustrated in Fig. 1.

### Pulsed-field gel electrophoresis.

In June 2017, at the beginning of this outbreak, routine WGS was not yet implemented in Canada for the surveillance of *Shigella* spp. PFGE was therefore initially used to investigate genetic relatedness according to PulseNet procedures (39). *Shigella* spp. isolates are sent to the Laboratoire de santé publique du Québec (LSPQ) on a voluntary basis for routine PulseNet surveillance. PFGE profiles and epidemiological data including time of collection, sex, and age of the patients were shared with the National Microbiology Laboratory (NML) for nationwide cluster detection.

### Whole-genome sequencing.

31 of the 33 S. sonnei isolates were retrospectively subjected to WGS once it was implemented as a routine PulseNet procedure. Two S. sonnei strains were not sequenced due to a failure to locate the isolates. Short-read sequencing with Illumina (Illumina Inc., San Diego, USA) was performed either at the NML or at the LSPQ. Sequencing protocols were performed as described elsewhere (40). The short reads of the 31 isolates have been deposited in the NCBI Sequence Read Archive under BioProject PRJNA639996. Illumina sequences were assembled using SPAdes in the BioNumerics v7.6 software (min contig length = 1,000). Resulting genome assemblies had a median of 361 contigs (range: 345–428).

### Phylogenetic analysis.

A reference-based core genome high-quality single-nucleotide variant (hqSNV) detection was performed using the SNVPhyl pipeline v.1.1 (41) integrated within the NML Galaxy platform (https://github.com/phac-nml/snvphyl-tools/) (42). The publicly available closed Shigella sonnei Ss046 chromosome (CP000038.1) was used for read mapping. PHASTER (43) and Island Viewer 4 (44) were used to identify prophage and genomic island sequences in the reference chromosome for mapping exclusion. Pipeline parameters and output reviewing were followed as per internal PulseNet standard operating procedures. A custom NML script (45) was used on the SNVPhyl maximum likelihood phylogenetic tree output to convert the branch length scale with an estimate of the number of hqSNV differences for its visualization in FigTree v1.4.3 (46).

Global phylogenetic reconstructions via the NCBI Pathogen Detection Isolates Browser (https://www.ncbi.nlm.nih.gov/pathogens/) enabled the identification of closely-related S. sonnei isolates submitted by other international public health agencies (accessed on 17 September 2021).

### Antimicrobial resistance and virulence predictions.

SPAdes assemblies were screened for acquired AMR genes and chromosomal point mutations using the ResFinder database with default parameters for E. coli (47, 48). Virulence genes were detected using the VirulenceFinder database with default parameters for E. coli (49).

### Data availability.

The fastq reads from all sequences analyzed in this article are available at the National Center for Biotechnology Information (NCBI) Short Read Archive (SRA) available at https://www.ncbi.nlm.nih.gov/sra under BioProject accession number PRJNA639996. Accession numbers can be found in Table 1.
